# Metabolomic Alteration in the Mouse Distal Colonic Mucosa after Oral Gavage with *Oxalobacter formigenes*

**DOI:** 10.3390/metabo10100405

**Published:** 2020-10-13

**Authors:** Casey A. Chamberlain, Marguerite Hatch, Timothy J. Garrett

**Affiliations:** Department of Pathology, Immunology, and Laboratory Medicine, University of Florida, Gainesville, FL 32610, USA; chamberlain.c@wustl.edu (C.A.C.); hatchma@pathology.ufl.edu (M.H.)

**Keywords:** *Oxalobacter formigenes*, metabolomics, mass spectrometry, liquid chromatography, oxalate, microbiome

## Abstract

*Oxalobacter formigenes* has been investigated for years due to its proposed ability to produce a secretagogue compound that initiates net intestinal oxalate secretion, thereby theoretically reducing circulating oxalate and risk of kidney stone formation. Strains which have been shown to exhibit this function in vivo across native tissue include the human strain, HC1, and the wild rat strain, OxWR. While previous work on these secretagogue-relevant strains has focused on profiling their metabolome and lipidome in vitro, efforts to characterize their influence on host intestinal mucosal biochemistry in vivo are yet to be reported. Much work has been done over the years with *O. formigenes* in relation to the secretagogue hypothesis, but it has never been clearly demonstrated that this microorganism is capable of inducing metabolic changes in native host tissue, which would be expected with the production of a transport-inducing compound. In this work, we show how the distal colonic mucosal metabolomic profile in a mouse model exhibited significant changes in the levels of a variety of metabolites as a result of oral gavage with *O. formigenes* HC1. Among these significant metabolites was nicotinic acid, an essential nutrient shown in past work to be produced in the gut by the native microbiome. Our finding that the in vivo biochemical state of the distal colon was altered with *O. formigenes* lends support to the secretagogue hypothesis and serves as a pioneering step in characterizing the biochemical interplay between *O. formigenes* and the mammalian host.

## 1. Introduction

The intestinal microbiome is estimated to contain thousands of different species of bacteria and each influences the health of the host in a unique manner [1]. One particular class of bacteria in the human gut microbiota is oxalotrophs (the “oxalobiome”) [2], which consume oxalate in the intestine [3]. Oxalate, a toxic byproduct of endogenous metabolism, as well as a ubiquitous component of many plant-based foods [4,5], is readily absorbed through the intestinal epithelium and precipitates with calcium in the kidneys to form calcium oxalate stones, representing approximately 80% of all diagnosed renal calculi [6]. Humans lack the enzymes needed to metabolize oxalate and are therefore dependent on their microbiome for its degradation and detoxification [7]. Perhaps the most well-studied intestinal oxalate degraders are *Oxalobacter formigenes* and select *Lactobacillus* and *Bifidobacterium* species [8]. When these bacteria break down oxalate in the intestine, the amount of free oxalate in the lumen that is available to be absorbed across the epithelium into the bloodstream is reduced, which theoretically lowers the risk of calcium oxalate stone formation [9]. *O. formigenes* is unique among oxalate degraders for two reasons: first, it relies exclusively upon oxalate as its sole energy source [10]; second, it has been shown to enhance enteric oxalate secretion by initiating a net oxalate flux into the intestinal lumen from the bloodstream [11]. This function allows *O. formigenes* to draw upon the endogenous circulating oxalate load as well as the exogenous dietary supply for increased substrate degradation. Hence, there is much interest in the oxalobiome, particularly *O. formigenes*, for its role in oxalate metabolism, kidney stone prevention, and potential applications to treatment of other oxalate diseases such as Primary Hyperoxaluria [8]. To date, the mechanism behind *O. formigenes* enhancing intestinal oxalate secretion remains uncharacterized. In their initial discovery of this function, Hatch et al. suggested that *O. formigenes* utilizes a secreted bioactive compound (a “secretagogue”) to stimulate transport of circulating oxalate into the lumen after it was shown that both *O. formigenes* (strain OxWR) colonization and the administration of an encapsulated *O. formigenes* lysate demonstrated this effect in the rat large intestine, but heat-treated whole cells and cell fragments failed to influence transport [11]. Building off their initial work, Hatch et al. subsequently showed stimulation of net intestinal oxalate secretion in the mouse large and small intestine using *O. formigenes* strain HC1, a human isolate [12]. With these compelling findings comes the notion that *O. formigenes* is perhaps capable of biochemically influencing the native intestinal mucosa to perform the secretagogue function, although this has never been clearly demonstrated to-date. If it were the case that *O. formigenes* can alter the intestinal metabolome, clarifying the nature of these induced molecular changes would be essential to understand the complete biochemical influence this microorganism exerts on the host colonic mucosa. To address this question, we tested the hypothesis that *O. formigenes* is capable of influencing the metabolome of the native intestinal mucosa by examining the chemical profiles of tissue from the mouse distal colon, an intestinal segment previously demonstrated to be acted upon by *O. formigenes* for oxalate secretion in both rats and mice [11,12], extracted from *O. formigenes*-gavaged and non-gavaged animals. Utilizing ultra-high-performance liquid chromatography–high-resolution mass spectrometry (UHPLC–HRMS), we analyzed and compared the mucosal metabolomes of these mice and observed notable biochemical divergence between gavaged and non-gavaged groups with alterations in the levels of a variety of metabolite features. Among these features is nicotinic acid (NA) which was significantly elevated (25.4-fold, *p* = 0.005) in the gavaged group. NA, also known as niacin or vitamin B3, is an essential nutrient whose dietary deficiency is associated with potentially life-threatening illness [13,14]. Hence, its elevation with *O. formigenes* gavage warrants further investigation regarding its potential impact on human health. While *O. formigenes* has been the focus of a variety of MS-based investigations [15,16,17], the novelty of this study is that it represents a metabolomic analysis of the interaction between *O. formigenes* and native host distal colonic tissue, which has never been reported. Findings provided by this work serve to increase our understanding of the biochemical footprint this microorganism exerts on the mammalian host.

## 2. Results and Discussion

### 2.1. Distal Colonic Metabolome Alteration with O. formigenes Oral Gavage

**Figure 1:**
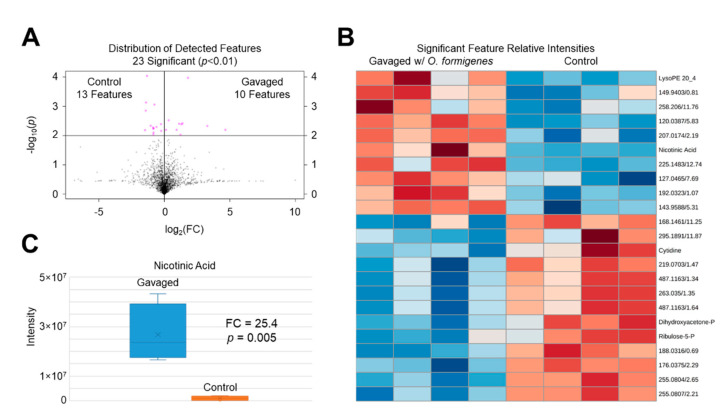
*Oxalobacter formigenes* oral gavage alters the mouse distal colonic mucosal metabolome. (**A**) Volcano plot showing detected features in the mouse distal colonic mucosa as a distribution of their statistical significance (*y*-axis) and fold change (*x*-axis). A total of 23 features were found to be significantly different (*p* < 0.01) with 13 elevated in the non-gavaged control group and 10 elevated in the gavaged group. (**B**) Heatmap showing the top 25 significant metabolite features between C57Bl/6 mice gavaged with 50 mg wet weight *O. formigenes* HC1 vs. non-gavaged mice (per group: *n* = 4; 2 males, 2 females). Data are Euclidean-distanced and Ward-clustered. Feature nomenclature: *m/z*/RT; “-P” indicates “Phosphate”. Red = elevated relative intensity, blue = reduced relative intensity. (**C**) Nicotinic acid is elevated (25.4-fold increase, *p* = 0.005) in the distal colonic mucosa of mice gavaged with *O. formigenes* relative to non-gavaged control mice.

The purpose of this study was to test the hypothesis that the intestinal mucosal metabolome could be altered by exposure to *O. formigenes*. Our findings confirm that mice gavaged with *O. formigenes* exhibited differential intestinal mucosal metabolic profiles than their non-gavaged counterparts. A volcano plot (Figure 1A) shows the distribution of all detected features by *p*-value and fold-change indicating the significant metabolic divergence observed. A panel of 23 features was found to have significant differences (*p* < 0.01) in signal intensities between the gavaged (10 elevated features) and control (13 elevated features) groups. A clear contrast between groups is observed when examining the relative intensity data for these significant features with a heatmap (Figure 1B).

As with any metabolomics experiment, the number of unidentified features usually far exceeds the number of those which are identified [18]. Among the 23 significant features, 5 (cytidine, dihydroxyacetone phosphate (DHAP), lysophosphatidylethanolamine (lysoPE) (20:4), nicotinic acid (NA), and ribulose-5-phosphate (R5P)) were identified by *m/z* and retention time (RT) matching to our method-specific metabolite library (level 1 identification confidence). Information for these 23 features is provided in Table 1. For the five identified metabolites, details regarding the detection and identification (*m/z*, RT, ion/adduct, mass accuracy, PubChem CID), significance (*p*-value, fold-change), and response to *O. formigenes* gavage (elevated or reduced signal intensity compared to the control) are shown. For the 18 unidentified metabolite features, information regarding their detection and annotation (putative formula assignment based on isotope analysis and/or *m/z* matching), significance, and response to *O. formigenes* are shown. For select unidentified features with sufficient signal intensity, chemical information gained from isotope analysis (# carbons, presence of sulfur, charge state) is presented. Mass spectra depicting isotope patterns for these select features are provided in Figures S1–S10. Assigned putative formulas and ions for these features agree with their predicated carbon count and charge state based on their associated isotope information. Formula assignment for unidentified species without isotope information was based solely on *m/z* matching with priority given to the singly-charged protonated species ([M + H]^+^) being the most commonly detected ion for most metabolites. If no formulas were returned for [M + H]^+^, the search was expanded to include other expected ions (e.g., [M + Na]^+^, [M + 2H]^2+^, etc.). Further work is needed to identify and characterize the 18 unknowns reported in this study, and their assigned putative formulas should currently be regarded as evidence-based possibilities to orient future investigations rather than as conclusive identities.

**Figure 2:**
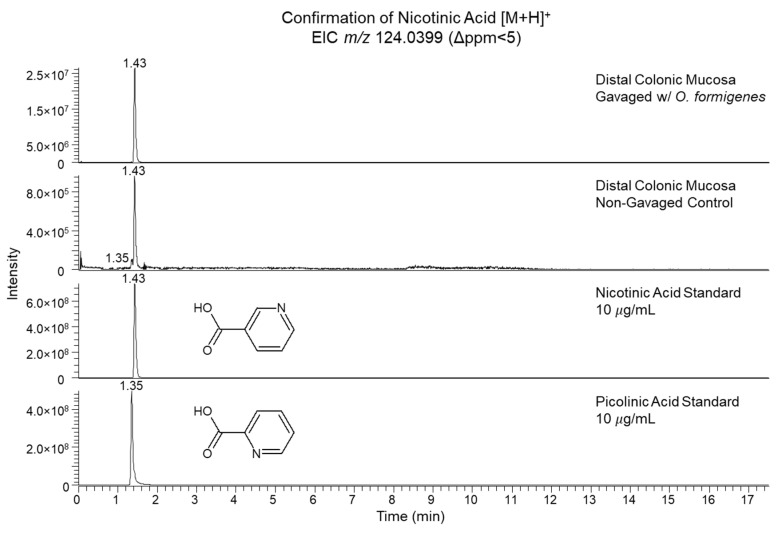
Nicotinic acid is confirmed in the distal colonic mucosa by analysis of pure standards by ultra-high-performance liquid chromatography–high-resolution mass spectrometry (UHPLC–HRMS). Extracted ion chromatogram for *m/z* 124.0399 (Δppm < 5) confirms identification of nicotinic acid by chromatographic alignment of *O. formigenes*-gavaged versus non-gavaged mouse distal colonic mucosal metabolite extracts against pure standards of nicotinic acid and picolinic acid. Peak of interest at 1.43 min in the distal colonic mucosa is identified as nicotinic acid by exact RT match to its pure standard and is differentiated from picolinic acid (1.35 min).

Our discussion will focus on the significant features which were identified. Cytidine, DHAP, and R5P were all found to be reduced in the gavaged distal colonic mucosa. R5P and DHAP are both key metabolites involved in primary carbon metabolism. DHAP is an intermediate in glycolysis formed from fructose-1,6-bisphosphate (F16BP) in the reversible reaction catalyzed by aldolase, which yields DHAP and glyceraldehyde-3-phosphate (G3P). DHAP has been long thought to act as a potential precursor for triglyceride (TG) biosynthesis [19,20,21], although the extent of its contribution to this pathway remains unclear and likely varies between tissue types [22]. Likewise, R5P is one of the end-products of the pentose phosphate pathway (PPP) formed from 6-phosphogluconate (6PG) by 6PG dehydrogenase [23]. A reduction in R5P pools could suggest differential flux and fates of PPP intermediates causing formation of end products to decrease. Two features were found to be elevated in the gavaged distal colonic mucosa: lysoPE (20:4) and NA. PEs represent a major lipid class in Gram-negative bacteria [24], as well as the most abundant lipid class in *O. formigenes* HC1 [15]. LysoPEs, derived from PEs, are known as lysophospholipids, a broad category of lipids which are missing one of its acyl chains, usually by enzymatic action by phospholipase A [25]. LysoPEs have been suggested to act as biologically active secondary messengers [26]. Hence, it is interesting that this specific lysoPE, one of many among a diverse profile of these lipids, was found to be elevated in the gavaged group, suggesting potential biological importance to *O. formigenes* in the gut. Among all the significant features identified, the most compelling was perhaps NA which was observed to have the greatest signal intensity difference by magnitude (25.4-fold elevation in gavaged group (*p* = 0.005)) (Figure 1C). NA, also known as niacin and one of the forms of vitamin B3 [13], was identified by *m/z* (Δppm < 1) and RT (1.43 min, ΔRT = 0.02 min) matching to our method-specific metabolite library. Upon observing that another structurally related compound in our metabolite library, picolinic acid (PA), shared the same chemical formula (and therefore the same accurate mass) and a RT difference < 0.1 min, we further confirmed the identity of NA by analyzing standards of both NA and PA and examining their aligned extracted ion chromatograms (*m/z* 124.0399, Δppm < 5) against the gavaged and non-gavaged mouse distal colonic mucosal extracts (Figure 2). NA showed an exact RT match (1.43 min) to the peak of interest in the intestinal extracts and a 0.08 min RT shift from PA (1.35 min). It appeared PA was detected at minimal levels in the distal colonic mucosa with a defined, low-abundance peak in the non-gavaged mouse, although no significant difference was detected between groups.

NA is regarded to be an essential vitamin to humans, meaning the primary method by which NA is obtained is through diet [14]. It is worth mentioning that NA can be synthesized to some extent in vivo from tryptophan [27], meaning cases of NA malnutrition are rarely seen in developed nations and have historically afflicted mainly poverty-stricken populations with low-protein diets [28]. Dietary deficiency of NA causes Pellagra, a disease commonly associated with the “4 D’s”, which are diarrhea, dermatitis, dementia, and death [13,29]. This pathology is believed to arise due to the disruption of a variety of metabolic pathways involving nicotinamide adenine dinucleotide, which requires NA as a biosynthetic precursor [30]. Our observation that oral gavage with *O. formigenes* significantly increased NA levels in the intestinal mucosa is interesting in that, whether directly (donation) or indirectly (stimulating production), this microorganism, or the microbiome in general, potentially promotes an enteric environment where this essential nutrient is supported, providing evidence for the first hypothesis of a theoretical non-oxalate-related health benefit from *O. formigenes*. The principle of a resident bacterium contributing necessary biochemicals to the mammalian host, specifically B vitamins, has been previously reported [31]; thus, our similar observation with *O. formigenes* and NA could possibly suggest new avenues for microbiome-based applications to NA malnutrition. However, being that *O. formigenes* is a minor component of the human intestinal microbiome, it remains unclear whether this effect would translate to humans. Significant further work is needed to fully understand this microbe-nutrient relationship.

For all metabolome changes associated with *O. formigenes* gavage discussed in this work, we emphasize that the nature of these observed differences remains unclear and deserves further investigation. While the purpose of this study was to determine whether *O. formigenes* could alter the intestinal metabolome, much work remains in characterizing the complete influence it exerts on the mammalian host, as well as the mechanism by which such changes are induced. There are many avenues of thought behind *O. formigenes* and its capability to alter the metabolome of the host distal colonic mucosa, a few possibilities being the following: (1) direct donation of specific metabolites via intracellular production followed by secretion to the mucosa; (2) indirect mucosal stimulus of metabolite biosynthesis by secretion of pathway-triggering signaling factors targeting the intestine; (3) indirect microbial stimulus of metabolite biosynthesis by secretion of signaling factors targeting other bacteria in the microbiome to produce specific metabolites or signaling factors to induce mucosal metabolite production; (4) alteration of the microbiome composition in a manner that favors colonization by bacteria which hold the capability to influence metabolite biosynthesis in the mucosa; (5) indirectly influencing the intestinal metabolome due to the microorganism’s effect on the oxalate load in vivo, which could affect the pooling or flux of other metabolites or induce changes in the microbiome with a metabolome-altering consequence. Significant future work is needed to clarify the biochemical nature and biological relevance of the metabolomic alterations associated with *O. formigenes* gavage.

### 2.2. Assessment of the Gavage Effect on Significant Features

To confirm the significance of these metabolite features was truly due to gavage with *O. formigenes* and not due to the act of gavage with the spent medium matrix in which it was delivered, a repeat experiment was conducted to compare the distal colonic mucosal metabolomes of non-gavaged control mice to a group gavaged with sterile spent medium (C57/BL6 mice (*n* = 3 per group)). *O. formigenes* from the same culture was grown in Medium B supplemented with 20 mM oxalate from the same batch as used in the original experiment. Immediately prior to gavage, *O. formigenes* from a 24-h culture was separated from the spent medium by pelleting the bacterial cells at 5000× *g* for 20 min at 4 °C, removing the supernatant, centrifuging the supernatant again at 5000× *g* for 20 min at 4 °C, and then removing and filter-sterilizing the supernatant twice using a 0.22 µm filter into a clean PP vial. All timelines involving animals, gavage, and diets were kept constant with the previous experiment, as were all parameters in tissue harvest, sample preparation, metabolite extraction, and UHPLC–HRMS analysis. The metabolomics results from the repeat experiment indicated no significant difference in the detection of any of the 23 metabolite features reported in this work. As an example, Figure S11 portrays the signal intensity data for NA in both experiments, showing no significant difference due to the act of gavage with spent medium. These results indicate that the act of gavage with spent medium matrix did not affect the metabolite features reported in this work and that their signal intensity differences are indeed due to gavage with *O. formigenes*.

## 3. Materials and Methods

### 3.1. Use of Animals

Use of mice in this study was in accordance with the University of Florida and the National Institutes of Health Guide for the Care and Use of Laboratory Animals. All protocols were approved by the University of Florida Institutional Animal Care and Use Committee (Protocol #201904003). All mice (C57Bl/6) were bred in the University of Florida Animal Care Facility. Mice were given free access to water and fed a standard chow TD.7912 (Harlan Teklad, Indianapolis, IN, USA) until recruitment into the experiment as adults aged 4–8 months. The purpose of their use in experimentation was to examine the effect of *O. formigenes* gavage on the metabolome of the distal colonic mucosa by comparing tissue removed from gavaged mice (*n* = 4) compared to non-gavaged mice (*n* = 4) by UHPLC–HRMS. Groups were balanced based on sex.

### 3.2. Gavaging and Tissue Harvest

For this investigation, all references to *O. formigenes* refer to the human isolate *O. formigenes* strain HC1.

Prior to experimentation, all mice used in this study were confirmed to be negative for baseline *O. formigenes* colonization using our previously reported fecal oxalate degradation assay [12]. Briefly, fecal content from each mouse was collected and inoculated into anaerobic media vials containing an *O. formigenes*-selective medium supplemented with 20 mM oxalate (Medium B, [10]). After incubating at 37 °C for 7 days, the oxalate concentration in the media vials was measured using an oxalate oxidase-based colorimetric assay (Trinity Biotech Plc, Bray, Co Wicklow, Ireland). Using this method, animals are determined to be colonized if the concentration of oxalate in the inoculated media vials is found to be significantly reduced compared to a non-inoculated control. In our experience, oxalate is completely depleted within 3–7 days incubation of *O. formigenes*-positive fecal inoculation.

Prior to esophageal gavage with *O. formigenes*, all mice were fed a 1.5% oxalate-supplemented diet (0.5% calcium/0.4% phosphate; diet D10001, Research Diets, Inc., NJ, USA) for 6 days in order to “prime” the gut for *O. formigenes*. *O. formigenes* was delivered by esophageal gavage with a 0.5 mL inoculum containing approximately 50 mg wet weight of bacteria from a 24-h culture suspended in the microbe’s spent medium (Medium B supplemented with 100 mM oxalate). The next day, the same mice were gavaged a second time in a similar manner, and the oxalate-supplemented diet for all mice was replaced by regular mouse chow (diet 7912; Harlan Teklad, Indianapolis, IN, USA). Mice were maintained on the regular diet under normal conditions for a period of 5 days to allow the luminal environment to normalize from oxalate supplementation. After 5 days, all eight mice (*n* = 4 gavaged, *n* = 4 non-gavaged) were euthanized by inhalation of 100% carbon dioxide followed by exsanguination via cardiac puncture. After euthanasia, distal colonic tissues were harvested from all animals simultaneously to ensure standardization of collection time, as well as handled expeditiously to both minimize and standardize the time tissues were ischemic, using the following process. Without delay, the entire distal colon was removed and opened as a flat sheet. Using the same oxalate assay described above [12], luminal fecal contents were removed and transferred to media vials to determine colonization status at the time of harvest. Tissues were thoroughly and rapidly cleansed of luminal fecal contents by washing in several large volumes of isotonic, chilled 0.9% saline solution. Excess saline was removed by blotting using a Kimwipe, and tissue segments were weighed and trimmed to 100 mg. Tissues were quickly transferred to 2 mL screw-capped cryo-resistant vials, then snap-frozen and held in liquid nitrogen until needed for metabolite extraction.

### 3.3. Pre-Extraction Sample Preparation

Tissue samples were removed from liquid nitrogen, thawed on ice, and transferred to 1.6 mL polypropylene (PP) vials containing 500 μL 5 mM ammonium acetate in water. Cubic zirconium beads (0.7 mm) were added to vials, and tissues were then homogenized using a FastPrep-96 bead beater (MP Biomedicals, Irvine, CA, USA) in 3–30 s pulses at 1800 rpm separated by brief incubations on ice to maintain a low temperature. Once homogenized, samples were centrifuged at 20,000× *g* for 20 min at 4 °C to pellet tissue debris, and 200 μL of the tissue homogenate supernatant was transferred to 1.6 mL PP vials. Although tissue samples were normalized to 100 mg, protein concentrations were measured using a Qubit 3.0 fluorometer (Thermo Fisher Scientific, Waltham, MA, USA) to ensure comparable homogenization efficiency, which showed no significant difference (relative standard deviation < 1%) in protein concentration between samples.

### 3.4. Metabolite Extraction

Samples were held on ice during all steps in the extraction process and all reagents were LC–MS grade (Thermo Fisher Scientific). Metabolites were extracted using a protein precipitation procedure similar to our previous work [32]. Briefly, to each 200 μL sample of tissue homogenate, 20 μL internal standard (IS) solution was added. Components of the IS mixture, dissolved in 0.1% formic acid in water, were as follows: Creatine-(1-methyl-D_3_), d-Leucine-D_10_, l-Tryptophan-D_3_, l-Tyrosine-^13^C_6_, l-Leucine-^13^C_6_, l-Phenylalanine-^13^C_6_, *N*-BOC-l-tert-Leucine, *N*-BOC-l-Aspartic Acid, Succinic Acid-D_4_, Salicylic Acid-D_6_, Caffeine-(1-methyl-D_3_) (each at 4 µg/mL), Propionic Acid-^13^C_3_ (8 µg/mL), l-Tryptophan-D_3_ (40 µg/mL) (Acros Organics, Fairlawn, NJ, USA). Samples were briefly vortexed after adding the IS mix, then 800 µL 8:1:1 acetonitrile:methanol:acetone was added for protein precipitation. Samples were vortexed and incubated on ice for 30 min. After incubation, samples were centrifuged at 20,000× *g* for 10 min at 4 °C to pellet the protein. Supernatants (750 μL) were transferred to new 1.6 mL PP vials, and dried under nitrogen at 30 °C. Once dried, samples were reconstituted in 100 μL 0.1% formic acid in water, vortexed, incubated on ice for 20 min, then centrifuged at 20,000× *g* for 10 min at 4 °C to pellet any remaining protein. Supernatants (80 μL) were removed and transferred to new 1.6 mL PP vials. From each vial, 10 μL was diluted with 90 μL 0.1% formic acid in water in glass LC–MS vials for analysis.

### 3.5. Analytical Instrumentation and Methodology

Samples were analyzed by UHPLC–HRMS on a Q Exactive mass spectrometer paired with a Dionex Ultimate 3000 UHPLC System (Thermo Fisher Scientific) using similar methodology to our previous work [33]. Briefly, metabolites were chromatographically separated on an ACE Excel 2 C18-pentafluorophenyl column (100 mm × 2.1 mm, 2.0 µm) (Advanced Chromatography Technologies, Ltd., Aberdeen, Scotland) by reverse phase gradient elution (Solvent A: 0.1% formic acid in water, Solvent B: acetonitrile) at 0.35 mL/min using the following method: 0–3 min: 100% A, 3–13 min: 100%→20% A, 13–16.5 min: 20% A, 16.5–20 min: 100% A at 0.6 mL/min (column flush and equilibration). Injection volume was 5 μL. Full scan data were acquired in positive ion mode at 35,000 mass resolution scanning from *m/z* 70–1000. Source settings were as follows: spray voltage: 3.5 kV; S-lens: 30%; sheath gas flow rate: 50; auxiliary gas flow rate: 10: sweep gas flow rate: 10; capillary temperature: 325 °C; auxiliary gas temperature: 350 °C.

### 3.6. Data Processing, Quality Control, and Statistical Analysis

Assessing the quality and reproducibility of our analysis, the relative standard deviation of internal standards was examined and verified to be <10% across all samples. The raw acquisition data in this study were processed in a similar manner to our previous work [15], which we describe in detail here. Files were converted from the native raw to the open source mzXML format using RawConverter [34]. MZmine 2 was used for all steps involved in peak picking and feature alignment including mass detection, chromatogram building, smoothing, chromatogram deconvolution, isotope peak grouping, join aligning, gap filling, duplicate peak filtering, and adduct/complex removal [35]. Metabolites were identified using our custom method-specific library by *m/z* (Δppm < 5) and RT (±0.15 min) matching. Putative chemical formulas for unidentified features were generated using METLIN [36] screening for *m/z* matches within 5 ppm to expected ions/adducts. Missing intensity values were replaced by half the minimum value in the dataset [37]. Signal intensities were normalized to total ion signal and autoscaled [38] using MetaboAnalyst 4.0 [39]. The two-tailed, unpaired Student’s *t*-test was used to determine statistical significance, which we define as *p* < 0.05.

## 4. Conclusions and Future Directions

This study demonstrated that the metabolomic profile of the mouse distal colonic mucosa was altered after oral gavage with *O. formigenes* with our observation of significant changes in the intensities of a panel of 23 metabolite features. The results from this work support the hypothesis that *O. formigenes* is capable of influencing the native intestinal mucosal metabolome, which aligns with the theoretical production of a bioactive “secretagogue” compound responsible for influencing the enteric epithelium to secrete oxalate. Future work should seek to clarify the nature and mechanisms behind the biochemical influence *O. formigenes* has on the gut mucosa, both in relation to the metabolites presented in this work and others yet to be investigated. Particular attention should be given to NA as the elevated intensity we found after *O. formigenes* gavage could suggest future probiotic treatments for malnutrition of this essential nutrient. An examination of the metabolomic influence of other *O. formigenes* strains which have demonstrated the ability to induce a net oxalate secretion across native epithelial tissue (such as OxWR) would allow for a more complete perspective regarding this microorganism’s relationship with the mammalian host.

## Figures and Tables

**Table 1:** Significant (*p* < 0.01) identified and unidentified metabolite features in the mouse distal colonic mucosa after oral gavage with *O. formigenes*. Identified metabolites: information regarding their detection and identification (*m/z*, RT, ion/adduct, mass accuracy, PubChem CID), significance (*p*-value, fold-change), and response to *O. formigenes* gavage (elevated or reduced signal intensity compared to the control) are shown. Unidentified metabolite features: information regarding their detection (*m/z*, RT, ion/adduct, mass accuracy), putative formula assignment (based on *m/z* matching within 5 ppm), significance (*p*-value, fold-change), and response to *O. formigenes* gavage (elevated or reduced signal intensity compared to the control) are shown. For select unidentified features, chemical information gained from isotope ratio analysis (# carbons, presence of sulfur, charge state) is presented. Assigned putative formulas and ions for these features agree with their associated isotope information.

Significant Identified Metabolites											
***m/z***	**Metabolite** (**PubChem CID**)		**ID Method**		**Chemical Formula** (**M**)	**Ion**	**Δppm**	**RT**	***p*-Value**	**FC**	**Response**
244.0925	Cytidine (6175)		*m/z*, RT		C_9_H_13_N_3_O_5_	[M + H]^+^	3.3	1.55	4.1 × 10^−3^	1.2	Reduced
171.0053	Dihydroxyacetone Phosphate (668)		*m/z*, RT		C_3_H_7_O_6_P	[M + H]^+^	3.5	0.74	4.6 × 10^−3^	2.1	Reduced
502.2925	LysoPE (20:4) (42607465)		*m/z*, RT		C_25_H_44_NO_7_P	[M + H]^+^	1.8	12.73	4.7 × 10^−3^	9.7	Elevated
124.0395	Nicotinic Acid (938)		*m/z*, RT		C_6_H_5_NO_2_	[M + H]^+^	3.2	1.43	6.3 × 10^−3^	25.2	Elevated
231.0261	Ribulose-5-Phosphate (439184)		*m/z*, RT		C_5_H_11_O_8_P	[M + H]^+^	3.9	0.78	9.1 × 10^−3^	1.8	Reduced
Significant Unidentified Metabolites											
***m/z***	(**^13^C/^12^C**) ×**100**	**# Carbons**	**Sulfur**	**Charge**	**Putative Formula** (**M**)	**Ion**	**Δppm**	**RT**	***p*-Value**	**FC**	**Response**
127.0465	9.89 ± 1.59	8.99 ± 1.44	Y	+2	C_8_H_16_N_2_O_5_S	[M + 2H]^2+^	1.6	7.69	9.4 × 10^−3^	2.4	Elevated
143.9588 ^a^	15.54 ± 8.27	14.13 ± 7.51	*n*	+1	Undetermined			5.31	4.1 × 10^−3^	2.5	Elevated
176.0375	5.71 ± 1.30	5.19 ± 1.18	Y	+1	C_6_H_9_NO_3_S	[M + H]^+^	0.6	2.29	8.8 × 10^−4^	1.7	Reduced
192.0323	7.48 ± 3.31	6.80 ± 3.01	*n*	+1	C_8_H_10_O_2_P	[M + Na]^+^	4.7	1.07	3.9 × 10^−3^	2.2	Elevated
219.0703	17.66 ± 4.85	16.06 ± 4.41	Y	+2	C_15_H_24_N_4_O_9_S	[M + 2H]^2+^	0.9	1.47	5.3 × 10^−3^	1.8	Reduced
255.0804 ^b^	22.85 ± 5.95	20.77 ± 5.41	Y	+2	C_18_H_28_N_4_O_11_S	[M + 2H]^2+^	2.4	2.65	9.2 × 10^−5^	2.5	Reduced
255.0807 ^b^	17.95 ± 6.93	16.32 ± 6.30	Y	+2	C_18_H_28_N_4_O_11_S	[M + 2H]^2+^	1.2	2.21	7.5 × 10^−4^	2.6	Reduced
263.0350	18.85 ± 2.29	17.14 ± 2.08	Y	+2	C_19_H_22_N_2_O_10_S_2_	[M + H + Na]^2+^	3.8	1.35	5.7 × 10^−3^	1.8	Reduced
487.1163 ^c^	18.97 ± 1.64	17.24 ± 1.50	Y	+1	C_16_H_30_N_4_O_5_S_4_	[M + H]^+^	1.8	1.34	5.8 × 10^−3^	1.8	Reduced
487.1163 ^c^	18.69 ± 2.18	16.99 ± 1.98	Y	+1	C_16_H_30_N_4_O_5_S_4_	[M + H]^+^	1.8	1.64	7.9 × 10^−3^	1.8	Reduced
120.0387	Undetermined				C_15_H_10_O_3_	[M + 2H]^2+^	0.8	5.83	1.1 × 10^−4^	3.5	Elevated
149.9403 ^a^	Undetermined							0.81	3.0 × 10^−3^	1.3	Elevated
168.1461 ^a^	Undetermined							11.25	6.5 × 10^−3^	2.8	Reduced
188.0316	Undetermined				C_6_H_9_N_3_S_2_	[M + H]^+^	2.7	0.69	1.4 × 10^−3^	2.7	Reduced
207.0174	Undetermined				C_4_H_6_N_4_O_4_S	[M + H]^+^	4.3	2.19	3.8 × 10^−3^	2.6	Elevated
225.1483	Undetermined				C_13_H_20_O_3_	[M + H]^+^	0.9	12.74	6.4 × 10^−3^	1.9	Elevated
258.2060	Undetermined				C_14_H_27_NO_3_	[M + H]^+^	1.5	11.76	6.4 × 10^−3^	1.1	Elevated
295.1891	Undetermined				C_17_H_26_O_4_	[M + H]^+^	4.4	11.87	5.1 × 10^−3^	1.5	Reduced

^a^ No formulas returned using METLIN or other generators based on *m/z* matching to expected ions/adducts within routine mass accuracy (5 ppm). ^b,c^ Distinct chemical species which are likely structurally similar due to similar *m/z* (Δppm = 1.2 ^b^, 0 ^c^), RTs (ΔRT = 0.44 ^b^, 0.3 ^c^ min), and fold-changes (ΔFC = 0.1 ^b^, 0 ^c^) between groups.

## Supplementary Materials

The following are available online at https://www.mdpi.com/2218-1989/10/10/405/s1, Figures S1–S10: Isotope ratio analysis for significant unidentified distal colonic mucosal metabolite features, Figure S11: Elevation of distal colonic mucosal nicotinic acid (NA) in mice gavaged with *O. formigenes* in spent media relative to the non-gavaged control is not due to the act of gavage with spent media matrix.
