# Supplementary material for: Metabolomic Alteration in the Mouse Distal Colonic Mucosa after Oral Gavage with Oxalobacter formigenes
Source: Metabolites. 2020 Oct 13;10(10):405. doi: 10.3390/metabo10100405 (PMC7650779; doi:10.3390/metabo10100405)
Supplement: Supplementary file 1 [file metabolites-10-00405-s001.pptx]

## Slide 1
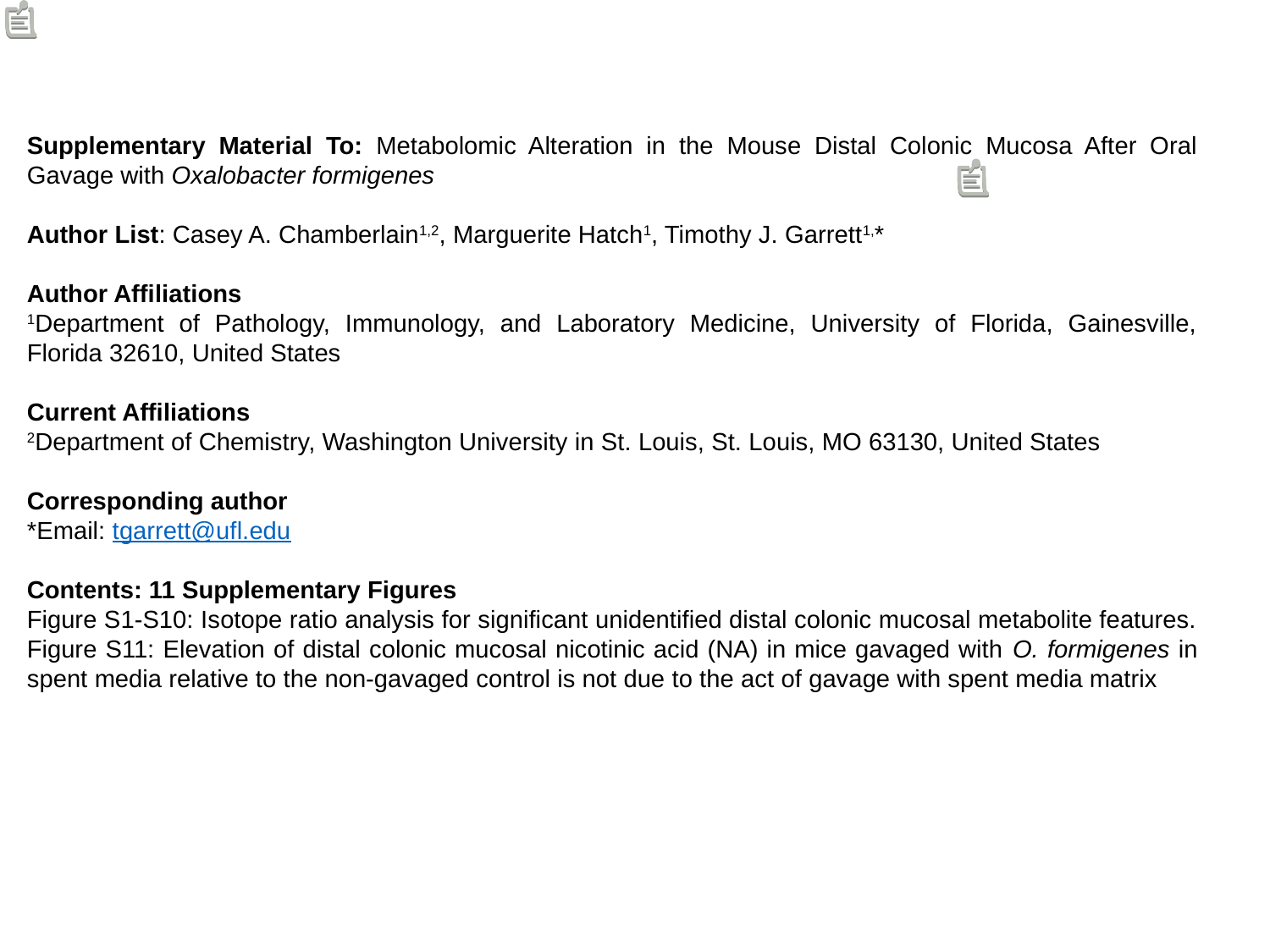

Supplementary Material To: Metabolomic Alteration in the Mouse Distal Colonic Mucosa After Oral Gavage with Oxalobacter formigenes
Author List: Casey A. Chamberlain1,2, Marguerite Hatch1, Timothy J. Garrett1,*
Author Affiliations
1Department of Pathology, Immunology, and Laboratory Medicine, University of Florida, Gainesville, Florida 32610, United States
Current Affiliations
2Department of Chemistry, Washington University in St. Louis, St. Louis, MO 63130, United States
Corresponding author
*Email: tgarrett@ufl.edu
Contents: 11 Supplementary Figures
Figure S1-S10: Isotope ratio analysis for significant unidentified distal colonic mucosal metabolite features. Figure S11: Elevation of distal colonic mucosal nicotinic acid (NA) in mice gavaged with O. formigenes in spent media relative to the non-gavaged control is not due to the act of gavage with spent media matrix

## Slide 2
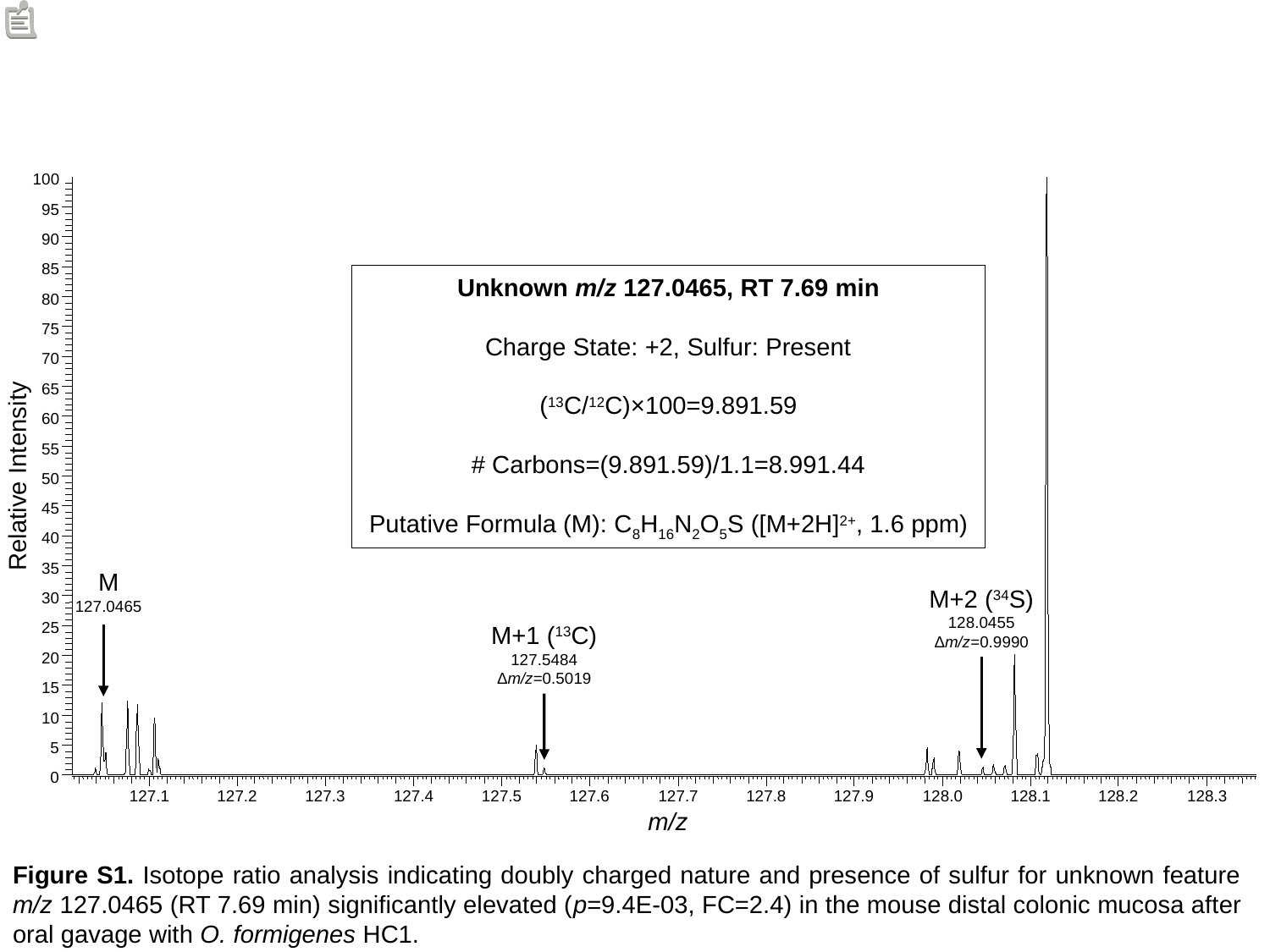

100
95
90
85
80
75
70
65
60
55
Relative Intensity
50
45
40
35
M
127.0465
M+2 (34S)
128.0455
Δm/z=0.9990
30
M+1 (13C)
127.5484
Δm/z=0.5019
25
20
15
10
5
0
127.1
127.2
127.3
127.4
127.5
127.6
127.7
127.8
127.9
128.0
128.1
128.2
128.3
m/z
Figure S1. Isotope ratio analysis indicating doubly charged nature and presence of sulfur for unknown feature m/z 127.0465 (RT 7.69 min) significantly elevated (p=9.4E-03, FC=2.4) in the mouse distal colonic mucosa after oral gavage with O. formigenes HC1.

## Slide 3
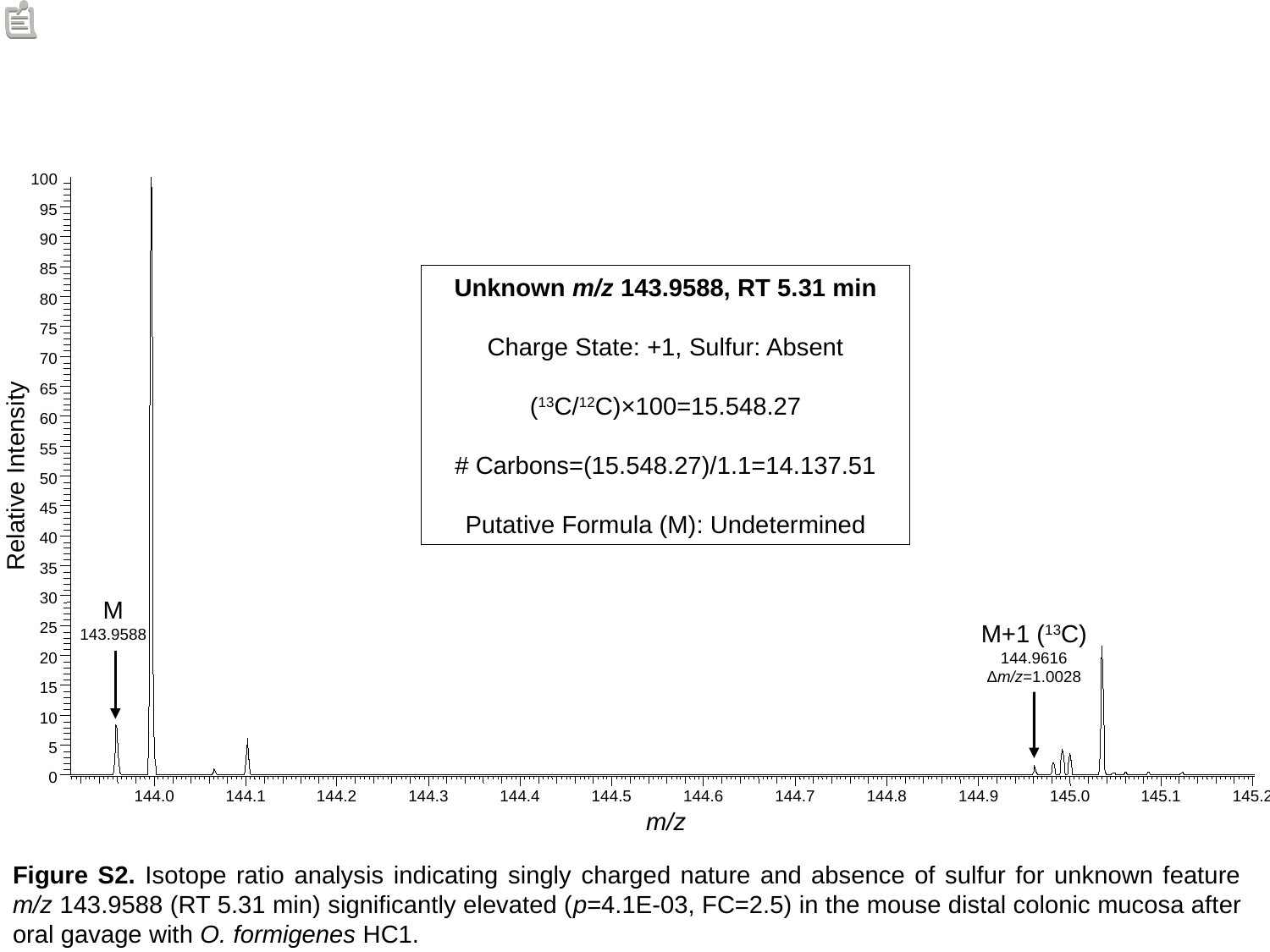

100
95
90
85
80
75
70
65
60
55
Relative Intensity
50
45
40
35
30
M
143.9588
M+1 (13C)
144.9616
Δm/z=1.0028
25
20
15
10
5
0
144.0
144.1
144.2
144.3
144.4
144.5
144.6
144.7
144.8
144.9
145.0
145.1
145.2
m/z
Figure S2. Isotope ratio analysis indicating singly charged nature and absence of sulfur for unknown feature m/z 143.9588 (RT 5.31 min) significantly elevated (p=4.1E-03, FC=2.5) in the mouse distal colonic mucosa after oral gavage with O. formigenes HC1.

## Slide 4
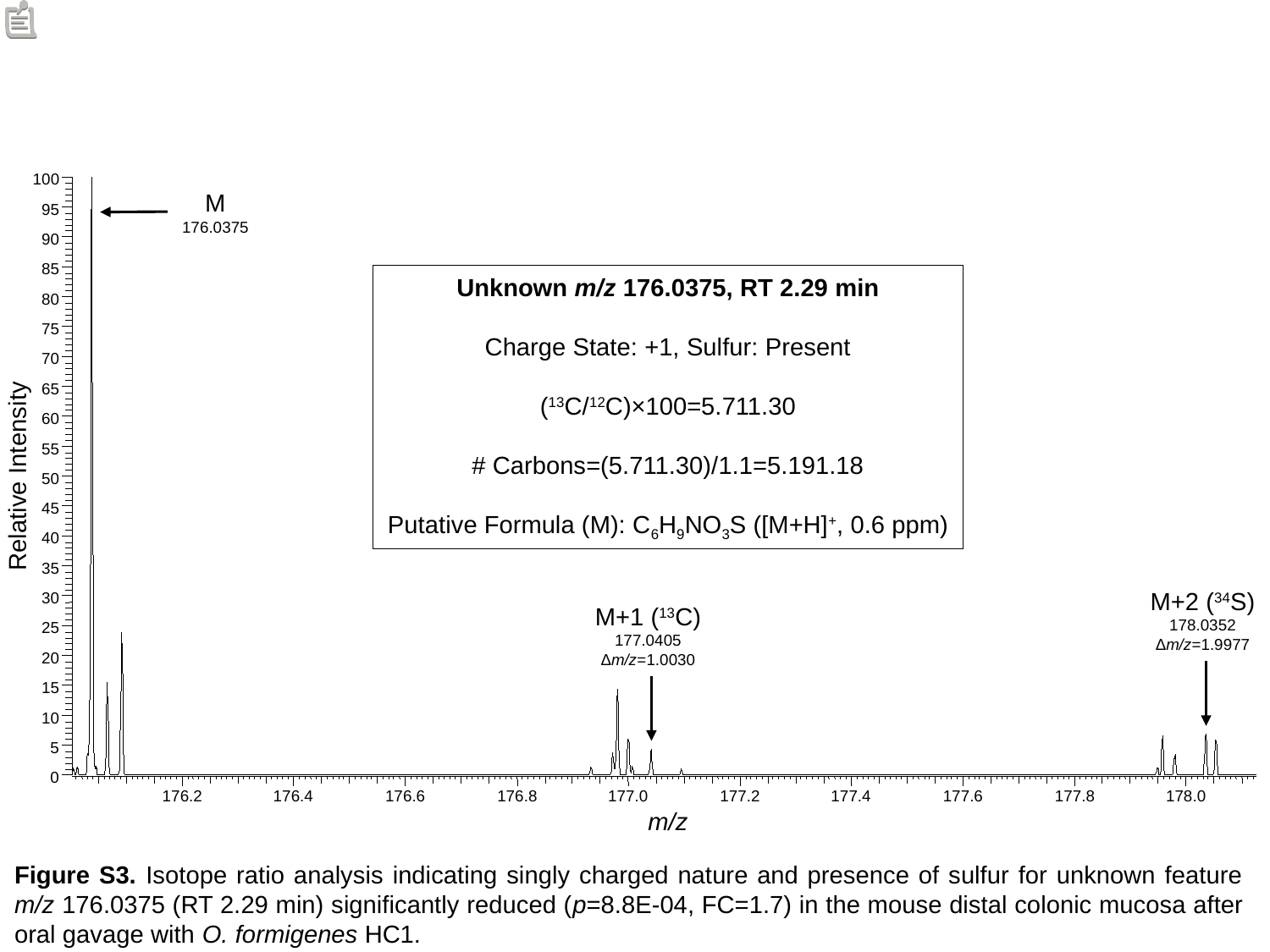

100
M
176.0375
95
90
85
80
75
70
65
60
55
Relative Intensity
50
45
40
35
M+2 (34S)
178.0352
Δm/z=1.9977
30
M+1 (13C)
177.0405
Δm/z=1.0030
25
20
15
10
5
0
176.2
176.4
176.6
176.8
177.0
177.2
177.4
177.6
177.8
178.0
m/z
Figure S3. Isotope ratio analysis indicating singly charged nature and presence of sulfur for unknown feature m/z 176.0375 (RT 2.29 min) significantly reduced (p=8.8E-04, FC=1.7) in the mouse distal colonic mucosa after oral gavage with O. formigenes HC1.

## Slide 5
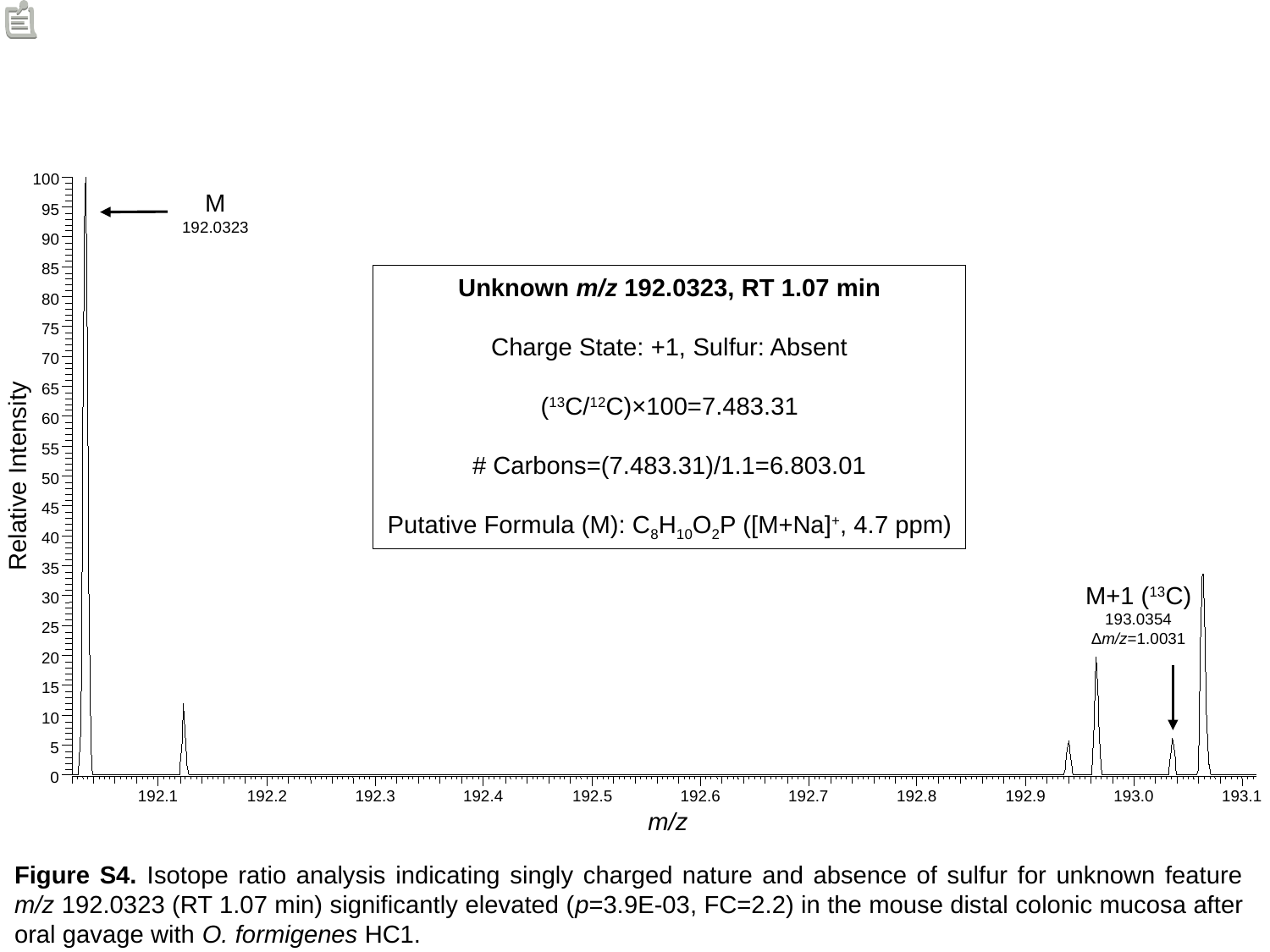

100
M
192.0323
95
90
85
80
75
70
65
60
55
Relative Intensity
50
45
40
35
M+1 (13C)
193.0354
Δm/z=1.0031
30
25
20
15
10
5
0
192.1
192.2
192.3
192.4
192.5
192.6
192.7
192.8
192.9
193.0
193.1
m/z
Figure S4. Isotope ratio analysis indicating singly charged nature and absence of sulfur for unknown feature m/z 192.0323 (RT 1.07 min) significantly elevated (p=3.9E-03, FC=2.2) in the mouse distal colonic mucosa after oral gavage with O. formigenes HC1.

## Slide 6
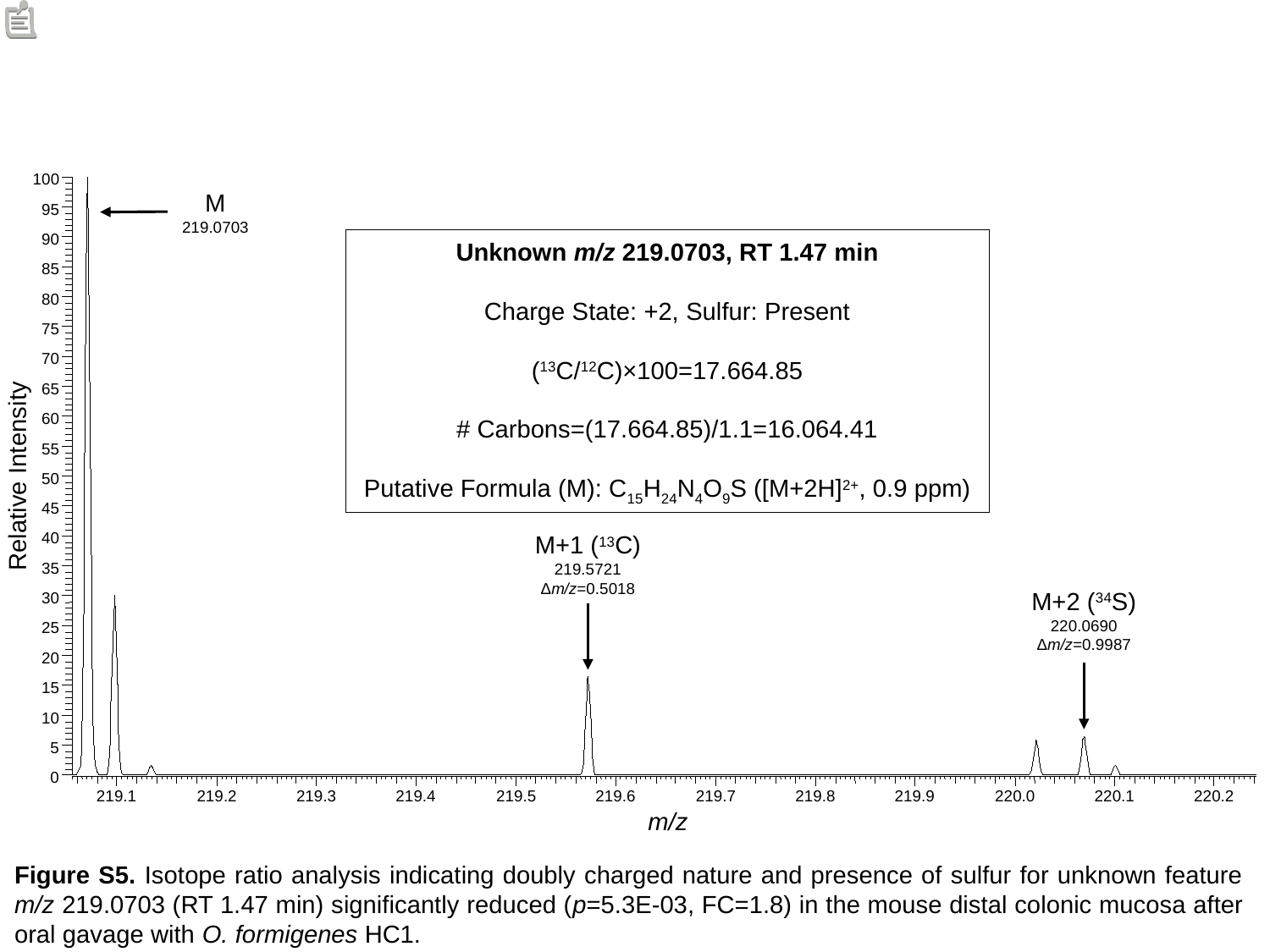

100
M
219.0703
95
90
85
80
75
70
65
60
55
Relative Intensity
50
45
M+1 (13C)
219.5721
Δm/z=0.5018
40
35
M+2 (34S)
220.0690
Δm/z=0.9987
30
25
20
15
10
5
0
219.1
219.2
219.3
219.4
219.5
219.6
219.7
219.8
219.9
220.0
220.1
220.2
m/z
Figure S5. Isotope ratio analysis indicating doubly charged nature and presence of sulfur for unknown feature m/z 219.0703 (RT 1.47 min) significantly reduced (p=5.3E-03, FC=1.8) in the mouse distal colonic mucosa after oral gavage with O. formigenes HC1.

## Slide 7
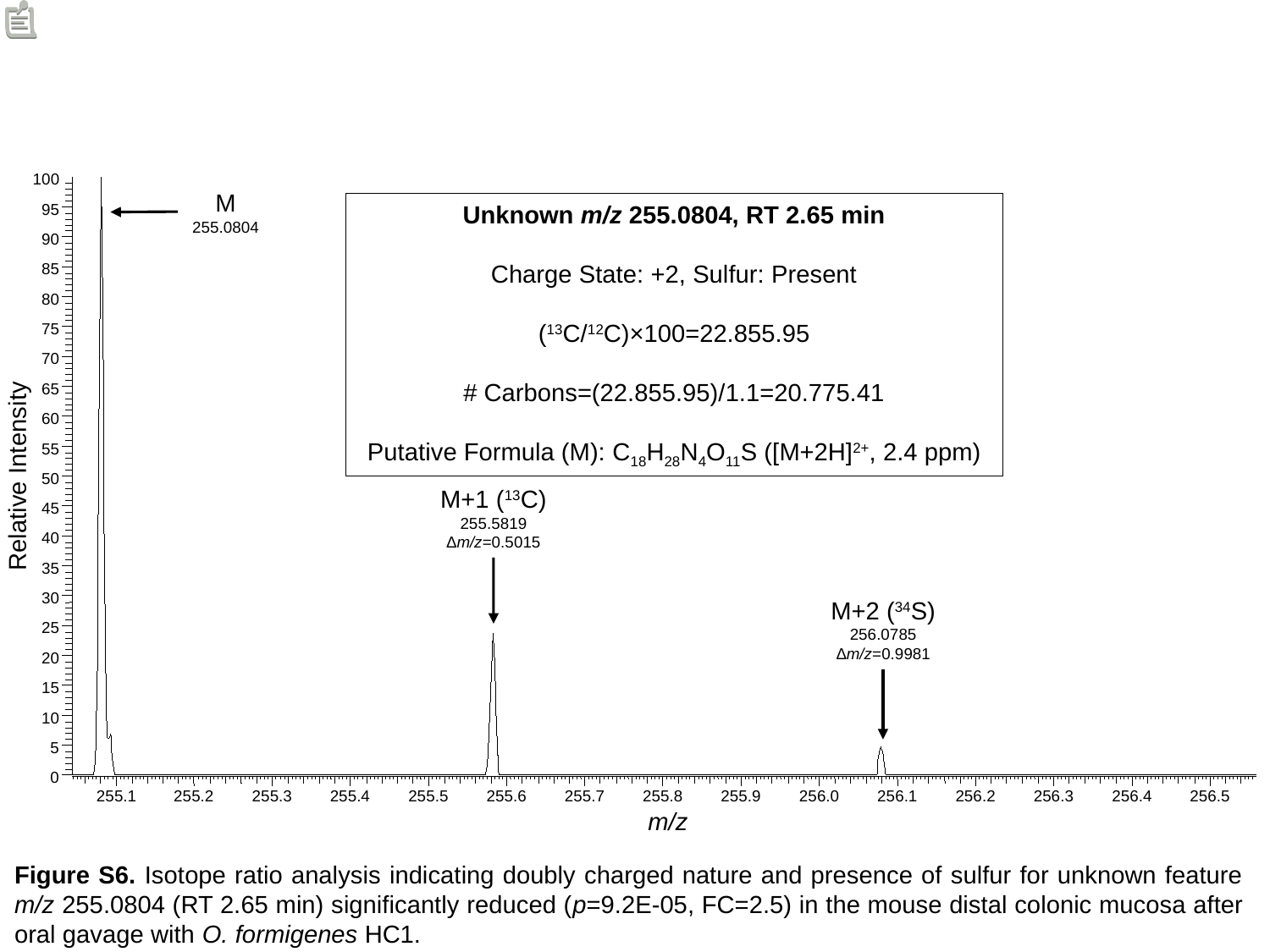

100
M
255.0804
95
90
85
80
75
70
65
60
55
Relative Intensity
50
M+1 (13C)
255.5819
Δm/z=0.5015
45
40
35
30
M+2 (34S)
256.0785
Δm/z=0.9981
25
20
15
10
5
0
255.1
255.2
255.3
255.4
255.5
255.6
255.7
255.8
255.9
256.0
256.1
256.2
256.3
256.4
256.5
m/z
Figure S6. Isotope ratio analysis indicating doubly charged nature and presence of sulfur for unknown feature m/z 255.0804 (RT 2.65 min) significantly reduced (p=9.2E-05, FC=2.5) in the mouse distal colonic mucosa after oral gavage with O. formigenes HC1.

## Slide 8
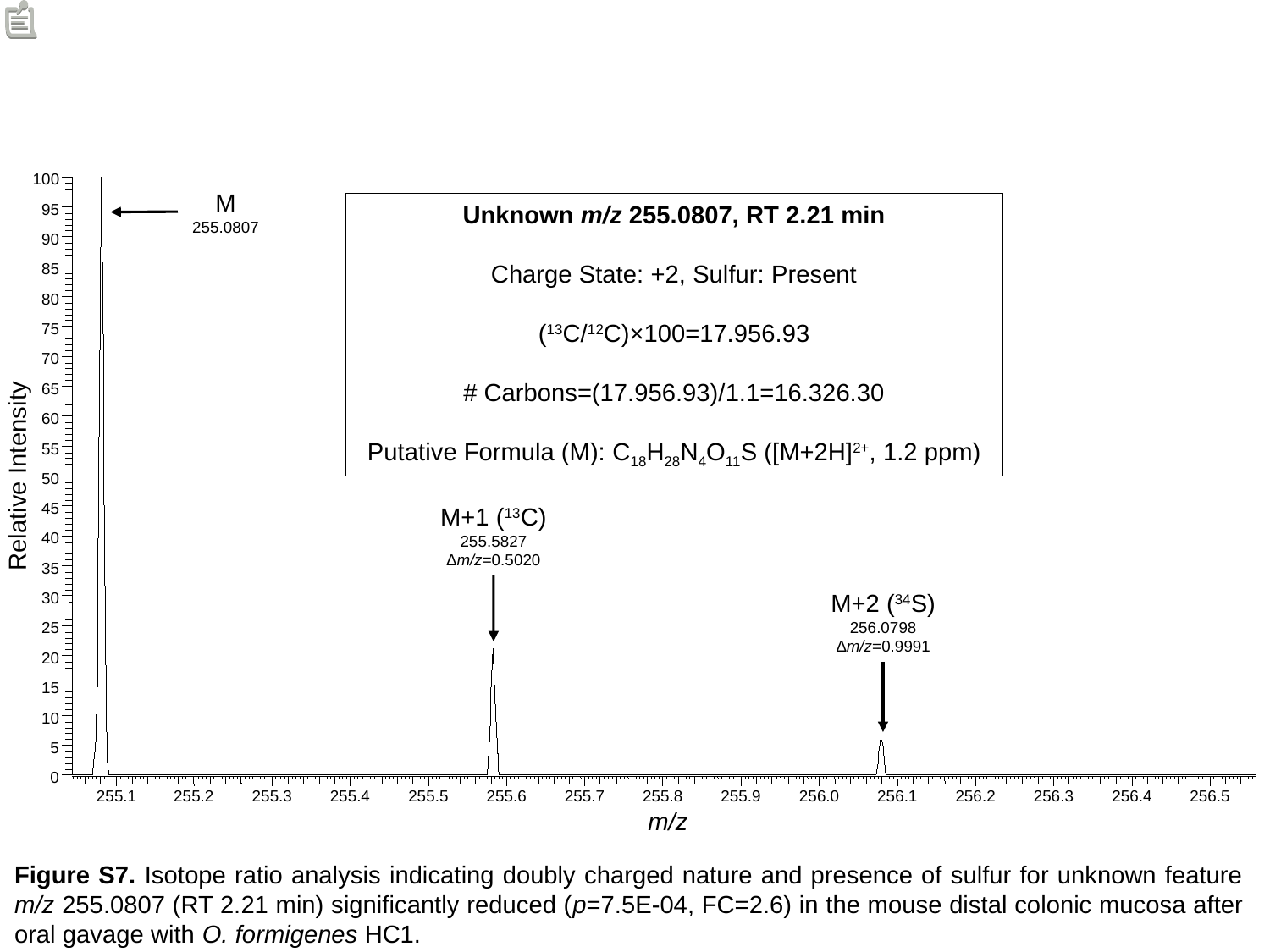

100
M
255.0807
95
90
85
80
75
70
65
60
55
Relative Intensity
50
M+1 (13C)
255.5827
Δm/z=0.5020
45
40
35
M+2 (34S)
256.0798
Δm/z=0.9991
30
25
20
15
10
5
0
255.1
255.2
255.3
255.4
255.5
255.6
255.7
255.8
255.9
256.0
256.1
256.2
256.3
256.4
256.5
m/z
Figure S7. Isotope ratio analysis indicating doubly charged nature and presence of sulfur for unknown feature m/z 255.0807 (RT 2.21 min) significantly reduced (p=7.5E-04, FC=2.6) in the mouse distal colonic mucosa after oral gavage with O. formigenes HC1.

## Slide 9
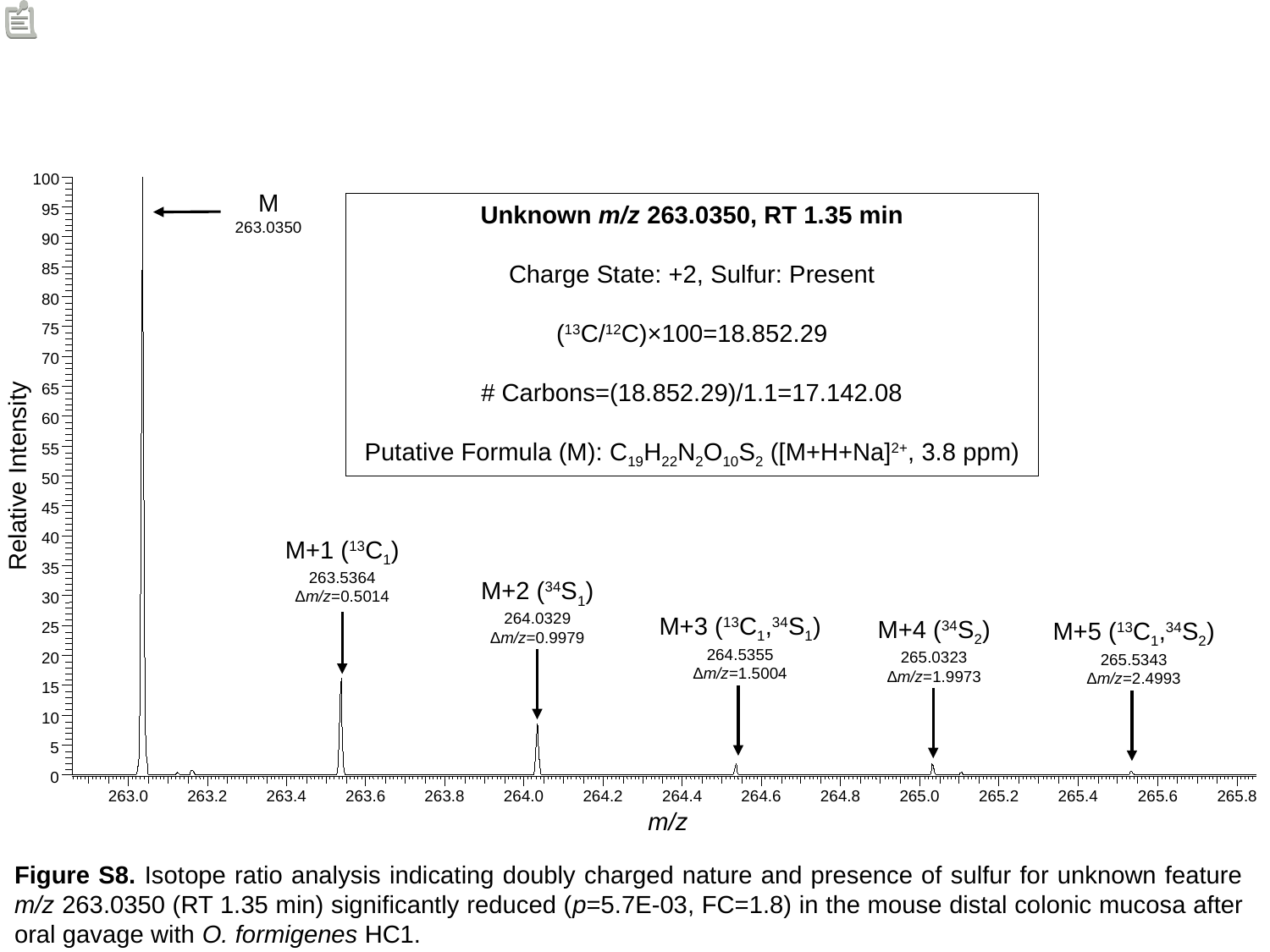

100
M
263.0350
95
90
85
80
75
70
65
60
55
Relative Intensity
50
45
M+1 (13C1)
263.5364
Δm/z=0.5014
40
35
M+2 (34S1)
264.0329
Δm/z=0.9979
30
M+3 (13C1,34S1)
264.5355
Δm/z=1.5004
M+4 (34S2)
265.0323
Δm/z=1.9973
M+5 (13C1,34S2)
265.5343
Δm/z=2.4993
25
20
15
10
5
0
263.0
263.2
263.4
263.6
263.8
264.0
264.2
264.4
264.6
264.8
265.0
265.2
265.4
265.6
265.8
m/z
Figure S8. Isotope ratio analysis indicating doubly charged nature and presence of sulfur for unknown feature m/z 263.0350 (RT 1.35 min) significantly reduced (p=5.7E-03, FC=1.8) in the mouse distal colonic mucosa after oral gavage with O. formigenes HC1.

## Slide 10
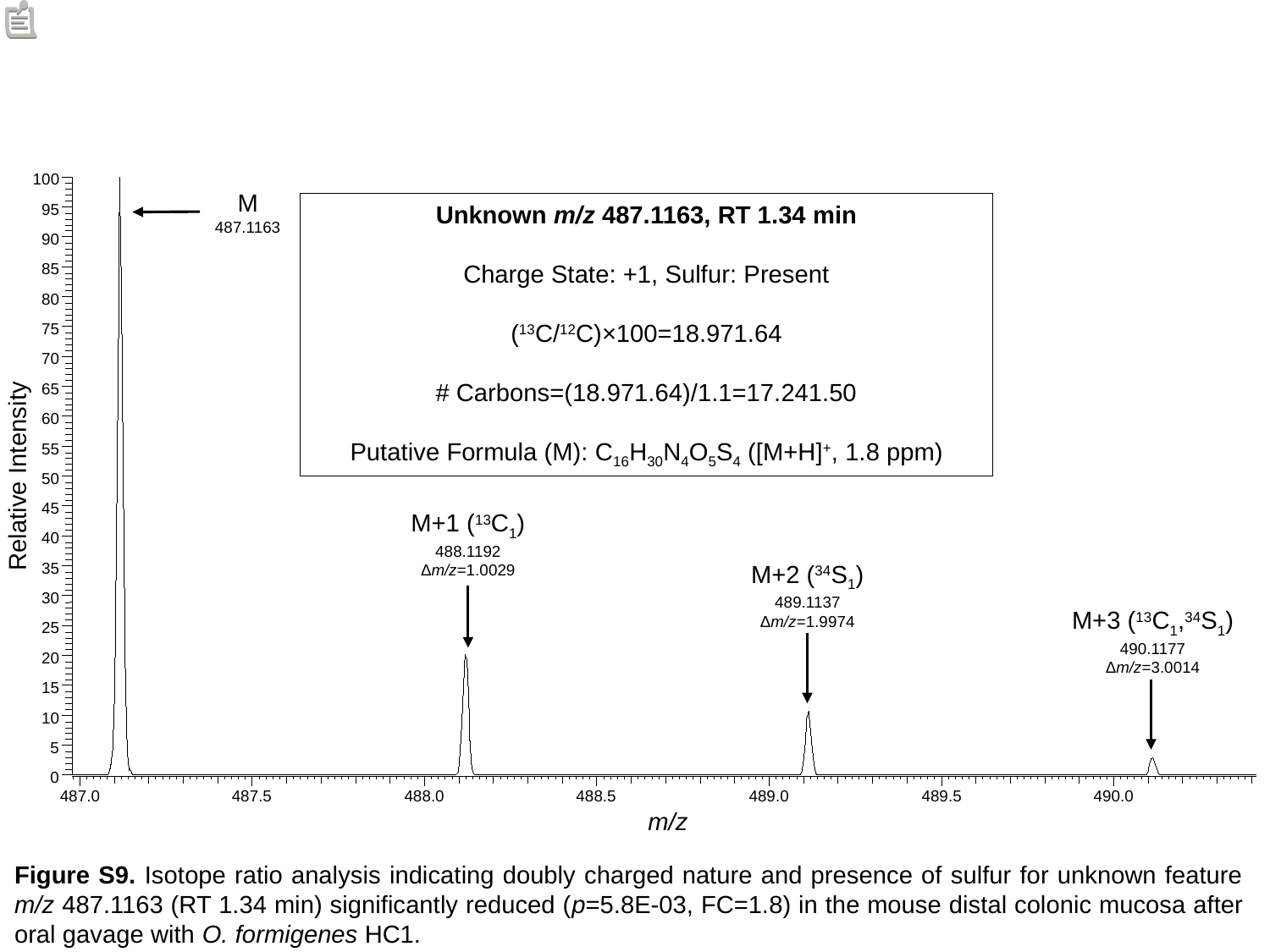

100
M
487.1163
95
90
85
80
75
70
65
60
55
Relative Intensity
50
45
M+1 (13C1)
488.1192
Δm/z=1.0029
40
M+2 (34S1)
489.1137
Δm/z=1.9974
35
30
M+3 (13C1,34S1)
490.1177
Δm/z=3.0014
25
20
15
10
5
0
487.0
487.5
488.0
488.5
489.0
489.5
490.0
m/z
Figure S9. Isotope ratio analysis indicating doubly charged nature and presence of sulfur for unknown feature m/z 487.1163 (RT 1.34 min) significantly reduced (p=5.8E-03, FC=1.8) in the mouse distal colonic mucosa after oral gavage with O. formigenes HC1.

## Slide 11
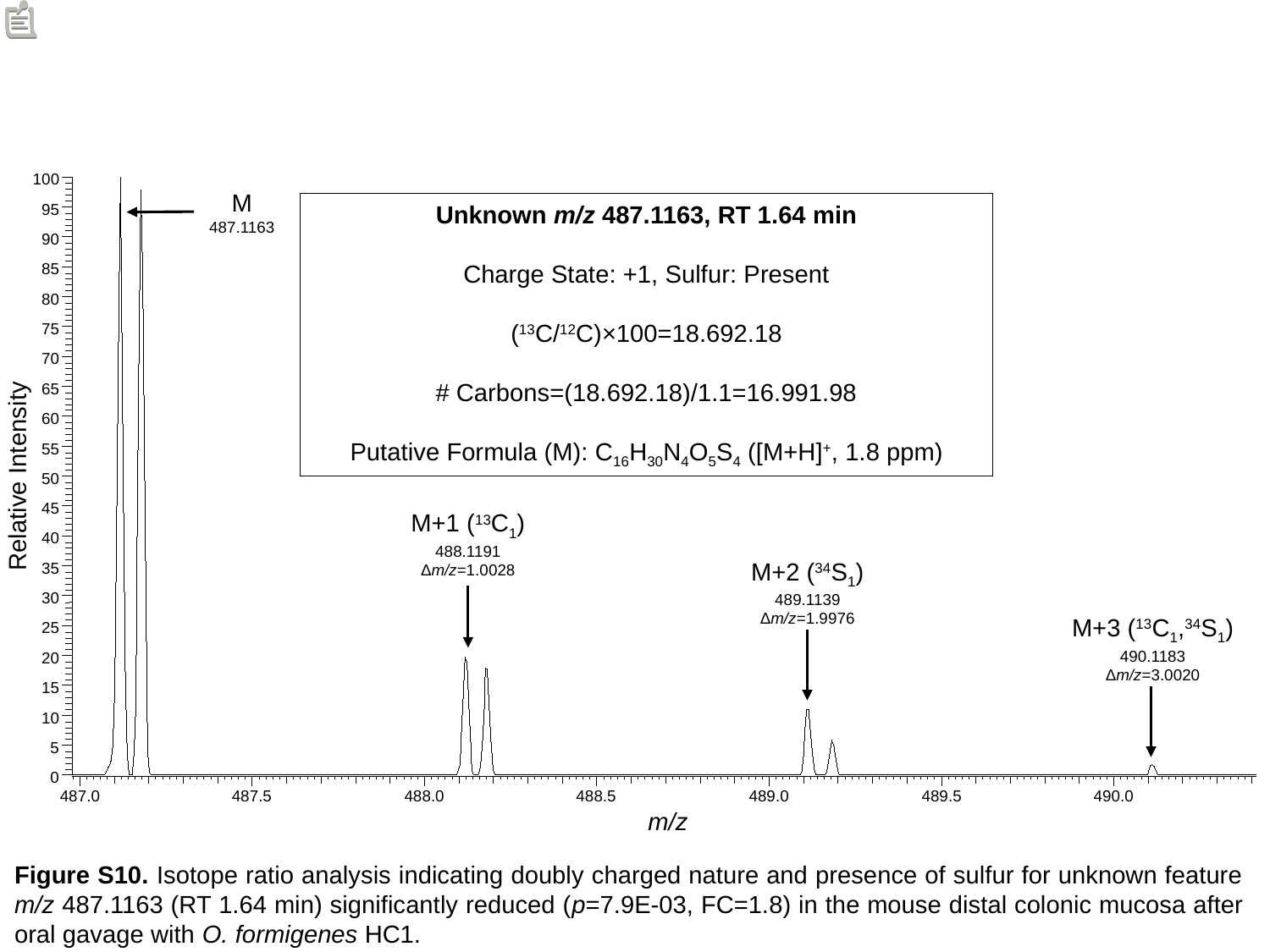

100
M
487.1163
95
90
85
80
75
70
65
60
55
Relative Intensity
50
45
M+1 (13C1)
488.1191
Δm/z=1.0028
40
M+2 (34S1)
489.1139
Δm/z=1.9976
35
30
M+3 (13C1,34S1)
490.1183
Δm/z=3.0020
25
20
15
10
5
0
487.0
487.5
488.0
488.5
489.0
489.5
490.0
m/z
Figure S10. Isotope ratio analysis indicating doubly charged nature and presence of sulfur for unknown feature m/z 487.1163 (RT 1.64 min) significantly reduced (p=7.9E-03, FC=1.8) in the mouse distal colonic mucosa after oral gavage with O. formigenes HC1.

## Slide 12
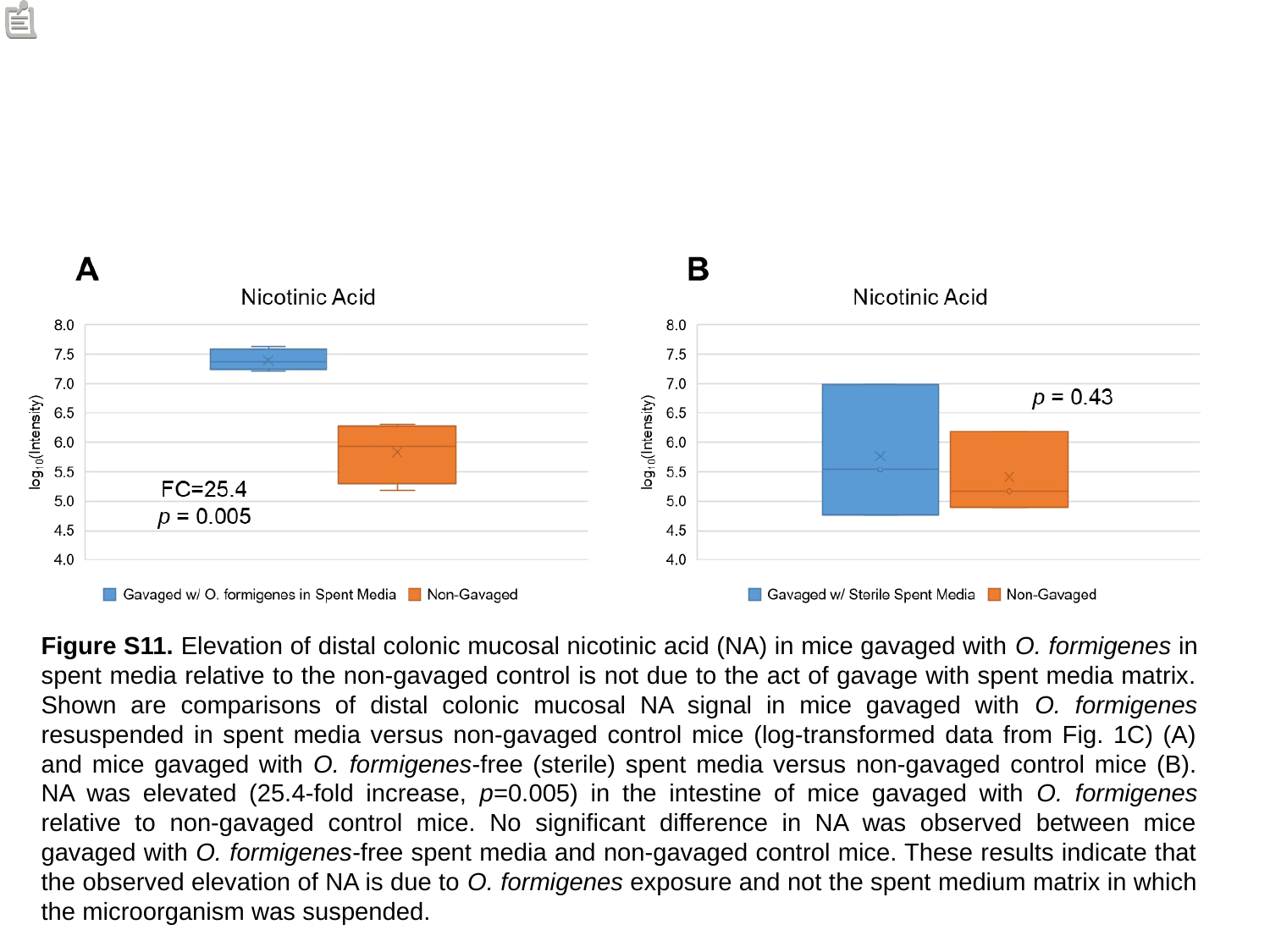

Figure S11. Elevation of distal colonic mucosal nicotinic acid (NA) in mice gavaged with O. formigenes in spent media relative to the non-gavaged control is not due to the act of gavage with spent media matrix. Shown are comparisons of distal colonic mucosal NA signal in mice gavaged with O. formigenes resuspended in spent media versus non-gavaged control mice (log-transformed data from Fig. 1C) (A) and mice gavaged with O. formigenes-free (sterile) spent media versus non-gavaged control mice (B). NA was elevated (25.4-fold increase, p=0.005) in the intestine of mice gavaged with O. formigenes relative to non-gavaged control mice. No significant difference in NA was observed between mice gavaged with O. formigenes-free spent media and non-gavaged control mice. These results indicate that the observed elevation of NA is due to O. formigenes exposure and not the spent medium matrix in which the microorganism was suspended.
